# Tongue paralysis after orotracheal intubation in a patient with primary mediastinal tumor: a case report

**DOI:** 10.1186/1757-1626-2-9301

**Published:** 2009-12-10

**Authors:** Esther Uña, Francisco Gandía, Jose Luis Duque

**Affiliations:** 1 Medical Oncology Service, Clinical University Hospital, Valladolid, Spain; 2 Intensive Care Unit, Clinical University Hospital, Valladolid, Spain; 3 Thoracic Surgery Service, Clinical University Hospital, Valladolid, Spain

## Abstract

**Introduction:**

Several lesions have been described as post-intubation complications. Most frequent are injuries of the pharynx/larynx or trachea. Cranial nerve injury following routine endo-tracheal intubation appears to be rare, and most reports describe Tapia's syndrome with hypoglossus/recurrent laryngeal nerve paralysis; cases that describe only bilateral hypoglossus palsy are infrequent. The cause is attributed to neuropathy of the nerve, provoked by compression following inflation of the cuff within the larynx or damage after neck hyperextension during a difficult intubation. However, similar cases after non-traumatic intubation have not been reported.

**Case presentation:**

We report here a case of bilateral hypoglossus palsy in a young man undergoing a diagnostic anterior mediastinotomy that was attributed to prolonged non-complicated oro-tracheal intubation. Progressive recovery of function by the patient supports neuropraxic damage as the cause.

**Conclusion:**

To avoid such problems, special attention should be paid to the correct positioning of the head during surgery or during rapidly performed tracheostomy if prolonged intubation is anticipated.

## Introduction

Several lesions have been described as post-intubation complications. Most frequent are injuries of the pharynx and larynx, such as oedema or ulcerations, chondromalacy of the larynx, oesophago-tracheal fistula, stenosis of the larynx or trachea and paralysis of the vocal cords [1]. Although the etiology of these injuries is not well known, it is believed that the chemical composition of the intubation tube or sterilization products used is the main cause. Pressure on adjoining tissues after oro-tracheal tube implantation has been described as another factor [1].

Few cases of hypoglossal nerve injury secondary to anaesthesia airway management have been reported. The cause of this type of lesion is attributed to neuropathy of the nerve provoked by compression following inflation of the cuff within the larynx or damage after hyperextension of the neck during a difficult intubation [2].

We report here a case of bilateral palsy of the hypoglossus nerve in a 28-year-old man undergoing a diagnostic anterior mediastinotomy as a part of an investigation of a mediastinal mass.

## Case Report

A 28-year-old white-man was referred to our hospital because he was complaining of a dry cough for 2 months; the cough had become associated with dyspnea in the week before he was referred to the hospital

He had previously been healthy with no history of smoking or alcohol intake. Physical examination revealed that, on thoracic auscultation, the heart and lung sounds were clearly audible. Chest imaging, including X-ray and computed tomography (CT), revealed a mass, measuring 15 × 12 × 10 cm, in the anterior mediastinum with pericardial and pleural effusions. Fine-needle puncture of the lesion revealed undifferentiated cells suggestive of lymphoma.

Diagnostic anterior mediastinotomy was performed on the patient to obtain a biopsy of the lesion and to complete the examination prior to the start of treatment.

Following fiberoptic tracheal intubation without sedation, general anaesthesia was administered. Diagnostic mediastinotomy was performed without incident, after which the patient exhibited peripheral desaturation to a level of 60%. The patient had to be intubated again and was kept in the intensive care unit.

Pathology revealed evidence of a yolk sac tumor, and laboratory results showed a marked elevation of serum alpha-fetoprotein (AFP) of up to 16780 ng/ml with normal serum levels of β-human chorionic gonadotropin. The patient received chemotherapy with EP (VP-16 or etoposide and cisplatin) without bleomycin to decrease the risk of pulmonary toxicity. As the patient showed very slow improvement, he had to undergo a tracheostomy to maintain tracheal intubation. Three courses of chemotherapy, adjusted to obtain the required dose per square meter (height, 182 cm; weight, 100 Kg), and 8 Gy of radiotherapy were necessary to reduce the tumor to a size sufficient for extubation and discharge to the hospitalization plant.

The tumor shrank to 10 × 7 × 5 cm and serum AFP dropped to 3750 ng/ml.

Overall, the patient remained a total of 70 days in the intensive care unit and was intubated for 62 days. After discharge, the patient complained of an inability to swallow and difficulty in speaking with normal vibration of the vocal cords. As a result, the patient lost 5 kg of weight and had to be fed through a nasogastric tube. Examination revealed no structural alterations of the soft palate. The tongue exhibited bilateral hypotrophy with an inability to move forward, suggesting bilateral hypoglossus palsy. Meticulous neurological examination, including magnetic resonance imaging (MRI) revealed no evidence of central or cranial nerve involvement.

Due to the spatial distance, we ruled out injury to the hypoglossal nerve caused by surgery. We attributed the cause of injury to neuropathy after prolonged compression of neighboring tissues by the tube, which was transferred to the nerve.

With conservative management, including steroids and speech-swallowing reeducation therapy, the patient achieved full recovery of lingual functions within four months after orotracheal intubation. Five months after the surgery, a progressive tumor in the anterior mediastinum was detected by chest CT. The patient received 4 additional courses of chemotherapy with TIP (ifosfamide, paclitaxel and cisplatin) and responded partially to this treatment. In the last follow-up, he was awaiting complete excisional surgery.

## Discusion

The hypoglossal nerve is a pure motor nerve that innervates all the muscles of the tongue. It is divided into five segments: medullary, cisternal, skull base, nasopharyngeal and oropharyngeal, carotid space, and sublingual. Each segment can potentially be affected by different disorders [3]. Atrophy or hypotrophy of tongue muscles is only seen when the nuclear or peripheral segments of the hypoglossal nerve are involved. In its position distal to the base of skull, the hypoglossal nerve may be affected by vascular aneurysm, local infection, surgical procedures such as carotid endarterectomy, accidental trauma or tumors [4].

There have been many cases of hypoglossal nerve palsy. In the Tommasi-Davenas [5]. series of 32 patients with paralysis of the hypoglossal nerve and tongue atrophy, only eight cases of isolated nerve XII palsy without neurologic involvement of other cranial nerves were identified; in most cases, the cause was a tumor, particularly a tumor arising from bone metastasis.

Few cases related to surgical procedure or anaesthesia airway management have been reported, and those that have been reported involved unilateral paralysis without tongue hypotrophy in combination with palsy of other cranial nerves, such as the recurrent laryngeal branch of the vague nerve (Tapia's syndrome) [6-9]. Although any surgical procedure can result in complications, ranging from mild to unexpected and severe episodes, this neuropathy is rare [10].

Gelmers (1983) described two cases of Tapia's syndrome after thoracotomy [10]. The cause was attributed to a structural lesion located at the point of crossing of the vagal and hypoglossal nerves. However, tongue paralysis could also have been due to neuropathy of both nerves as a result of inflation of the cuff within the larynx with secondary neuropathy. Alternatively, damage to the nerves after traction of the esophagus and transfer of the damage to both nerves which are closely connected at many sites, could be the cause

Yavuzer at al. (2004) described another case of Tapia's syndrome after septorhinoplasty. They believed that the condition was caused by pressure-induced neuropathy of the nerves due to inflation of the cuff within the larynx [11].

After a careful review of the literature, two probable mechanisms of nerve injury are likely: compression by the endo-tracheal tube following application of a throat pack to the oropharynx or a stretching mechanism of the nerves due to excessive flexion of the head [2]. These two mechanisms of nerve injury are believed to be of neuropraxic origin, which is likely to result from pressure on the lateral roots of the tongue during routine transoral intubation [2,12,13]. Another case of Tapia's syndrome following direct injury of the soft palate and oro-pharynx by a foreign body has been described [2].

Our patient presented with clinical signs that helped us localize the lesion to the peripheral segment of cranial nerve XII. Neuroimaging revealed no lesions in the central nervous system or cranial nerves. Although not a case of complete Tapia's syndrome, we describe our case as bilateral incomplete Tapia's syndrome with isolated bilateral paralysis of the muscles of the tongue after hypoglossus damage, without evidence of other affected cranial nerves.

In the current case, the clinical picture cannot be directly ascribed to the surgical procedure or tumor compression because of the spatial distance between the surgical or tumor site and the hypoglossus nerve and its branches. We attribute the clinical course to neuropathy following prolonged oro-tracheal intubation, even though it was not difficult or complicated.

There are very few cases in the literature describing cases similar to the current one, where postoperative bilateral Tapia's syndrome with isolated hypoglossal paralysis without other nerve involvement occurred after airway management in a surgical procedure with oro-tracheal intubation [8].

The progressive recovery of function by our patient and in the majority of cases reported in the literature support a neuropraxic type of nerve damage [2].

Although cranial nerve injury following routine endo-tracheal intubation appears to be rare, this complication should be considered by the otolaryngologist and anaesthesiologist [2]. Special attention should be paid to correct positioning of the head during surgery to avoid such problems and early initiation of language/swallowing reeducation is advisable [2].

## Consent

Written informed consent was obtained from the patient for publication of this case report. A copy of the written consent is available for review by the Editor-in-Chief of this journal.

## Competing interests

The authors declare that they have no competing interests.

## Authors' contributions

EU, FG and JLD contributed to the literature review, case reporting and manuscript revisions. EU drafted and edited the manuscript, and contributed to the final revision and approval.
